# A Rare Case Study About Necrotizing Granulomatous Sarcoidosis

**DOI:** 10.7759/cureus.10220

**Published:** 2020-09-03

**Authors:** Azka Tasleem, Hamza Viquar, Haris Noorani, Ravi Savani, Anchit Bharat

**Affiliations:** 1 Internal Medicine, Ball Memorial Hospital, Muncie, USA; 2 Pulmonology and Critical Care, Ball Memorial Hospital, Muncie, USA; 3 Internal Medicine, Indiana University Health Ball Memorial Hospital, Muncie, USA

**Keywords:** necrotizing sarcoid granulomatosis, sarcoid, high-value care

## Abstract

Sarcoidosis is a granulomatous disorder with an elusive etiology and pathogenesis. Classically, sarcoidosis is associated with non-caseating granulomas composed of mononuclear phagocytes, lymphocytes, and multinucleated giant cells. Necrotizing granulomas can also be associated with sarcoidosis but is scarcely reported in the medical literature. Necrotizing sarcoid granulomatosis is challenging to diagnose due to its rarity and similarity with other necrotizing disorders. Therefore, it is mainly considered a diagnosis of exclusion. We report one such case study, which could prompt further research to lay the course of treatment strategies for this disease. Moreover, our patient had a family history of sarcoidosis, which raises questions regarding possible genetic predisposition, and future work might help solve this medical mystery.

## Introduction

Sarcoidosis is a disease that usually involves various systems of the body. Approximately 90% of patients who present with sarcoidosis have significant lung manifestations, including but not limited to hilar lymphadenopathy, reticular opacities, and parenchymal nodules on imaging [1,2]. The annual incidence of sarcoidosis is not well known. However, it is more common in African Americans (females more than males) compared to Caucasians [3]. The diagnosis is suspected on the basis of clinical or radiographic findings and confirmed by the presence of non-caseating granulomas surrounded by multinucleated giant cells and lymphocytes on histopathological examination [4], after excluding other infectious and non-infectious causes that might present with similar findings, such as bacterial, fungal, parasitic and viral infections, chronic granulomatous disease, lymphoma, drug-induced granulomas, foreign body granulomatosis, and chronic granulomatosis with polyangiitis (GPA). Necrotizing sarcoid granulomatosis (NSG) is a rare variant of sarcoidosis, first described by Averill Abraham Liebow in 1973 [5]. However, only a few cases have been reported to date. This rare entity's diagnostic criteria include exclusion of other disorders with necrotizing granulomas, mainly GPA, rheumatoid nodule, tuberculosis (TB), and parasitic and fungal infections [6]. Here we discuss a case of NSG in a 29-year-old male who had an incidental finding of hilar, mediastinal, and abdominal lymphadenopathy on imaging. Hence, the focus is to highlight how sarcoidosis, in addition to being a multisystem disorder, can also manifest as rare histological features. This knowledge of rare variants of this disease process can help make a timely diagnosis and provide high-value care to the patients. 

## Case presentation

**Figure 1:**
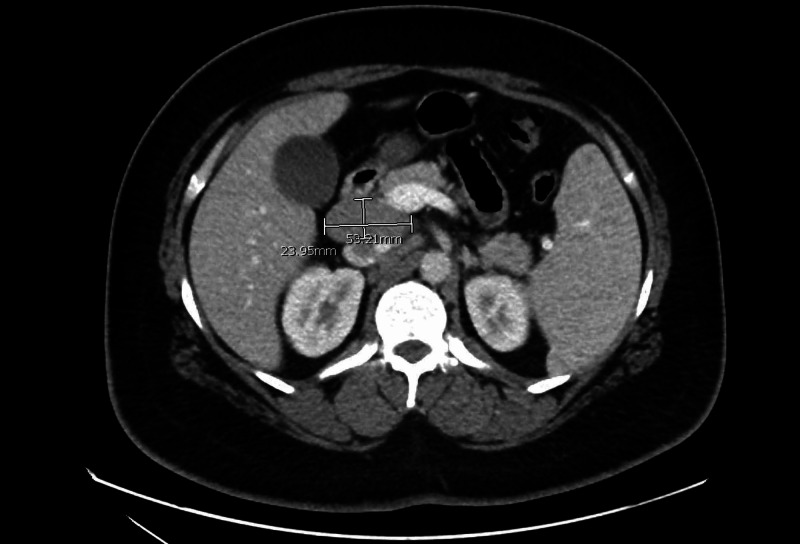
CT abdomen showing a transverse view of enlarged periportal lymph nodes measuring 2.4 cm × 5.3 cm in largest dimension (marked in white).

**Figure 2:**
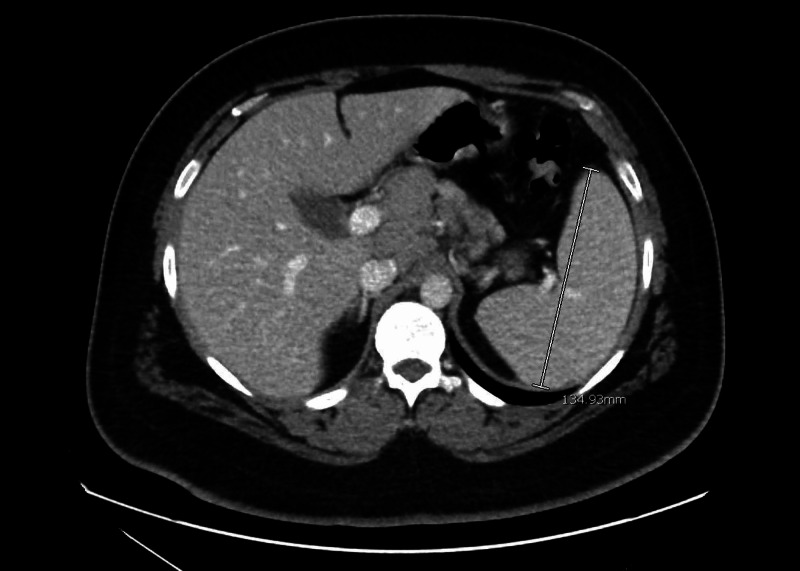
CT abdomen transverse section showing enlarged spleen measuring 13.49 cm in largest dimension (marked in white).

A 29-year-old male with a past medical history of seasonal allergies and back pain after a motor vehicle accident presented to the emergency department (ED) due to a week of multiple episodes of vomiting and epigastric pain, attributed to polypharmacy. In the ED, he received hydrocodone-acetaminophen and a bolus of normal saline. Workup included a CT of the abdomen, which showed splenomegaly and enlarged periportal and gastrohepatic lymph nodes (Figures 1, 2).

**Figure 3:**
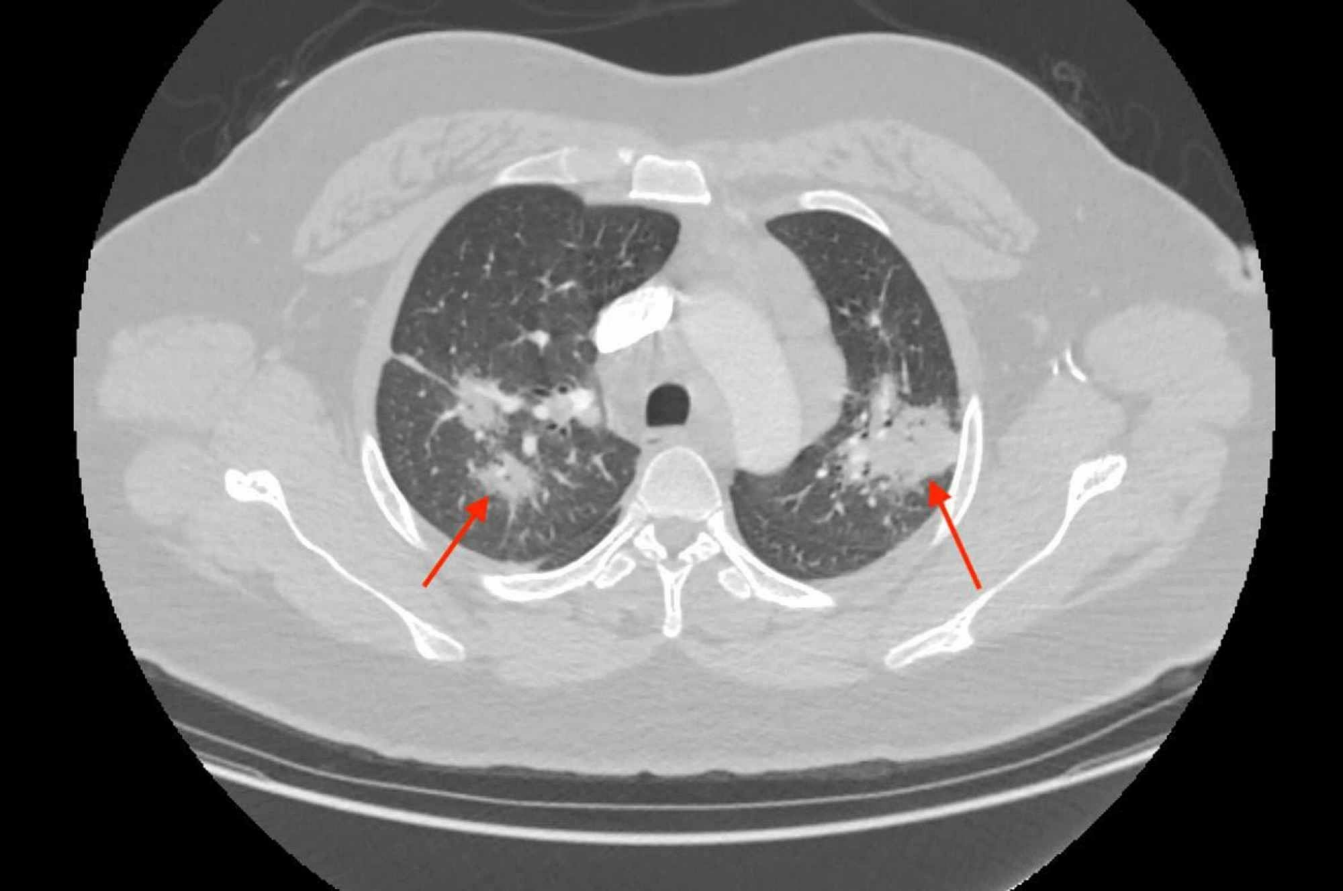
CT angiography chest transverse view showing bilateral pulmonary infiltrates.

**Figure 4:**
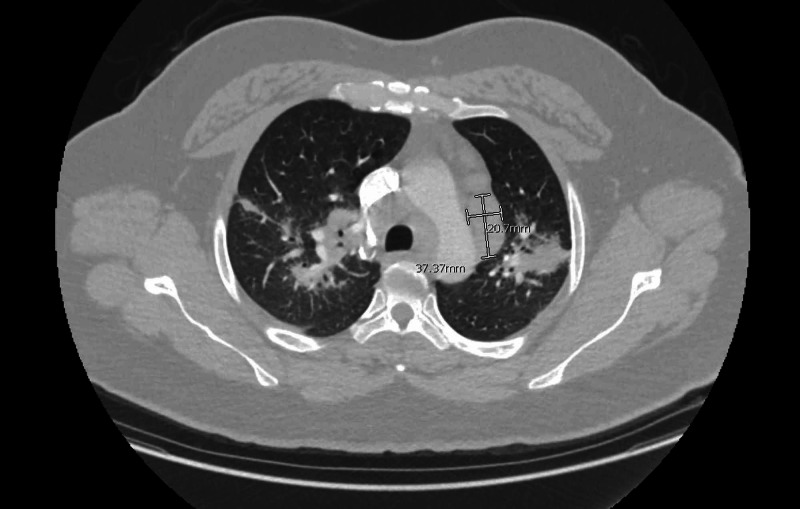
CT angiography chest transverse view showing enlarged left anteroposterior lymph node measuring 2.1 cm × 3.7 cm (dimensions marked in white).

**Figure 5:**
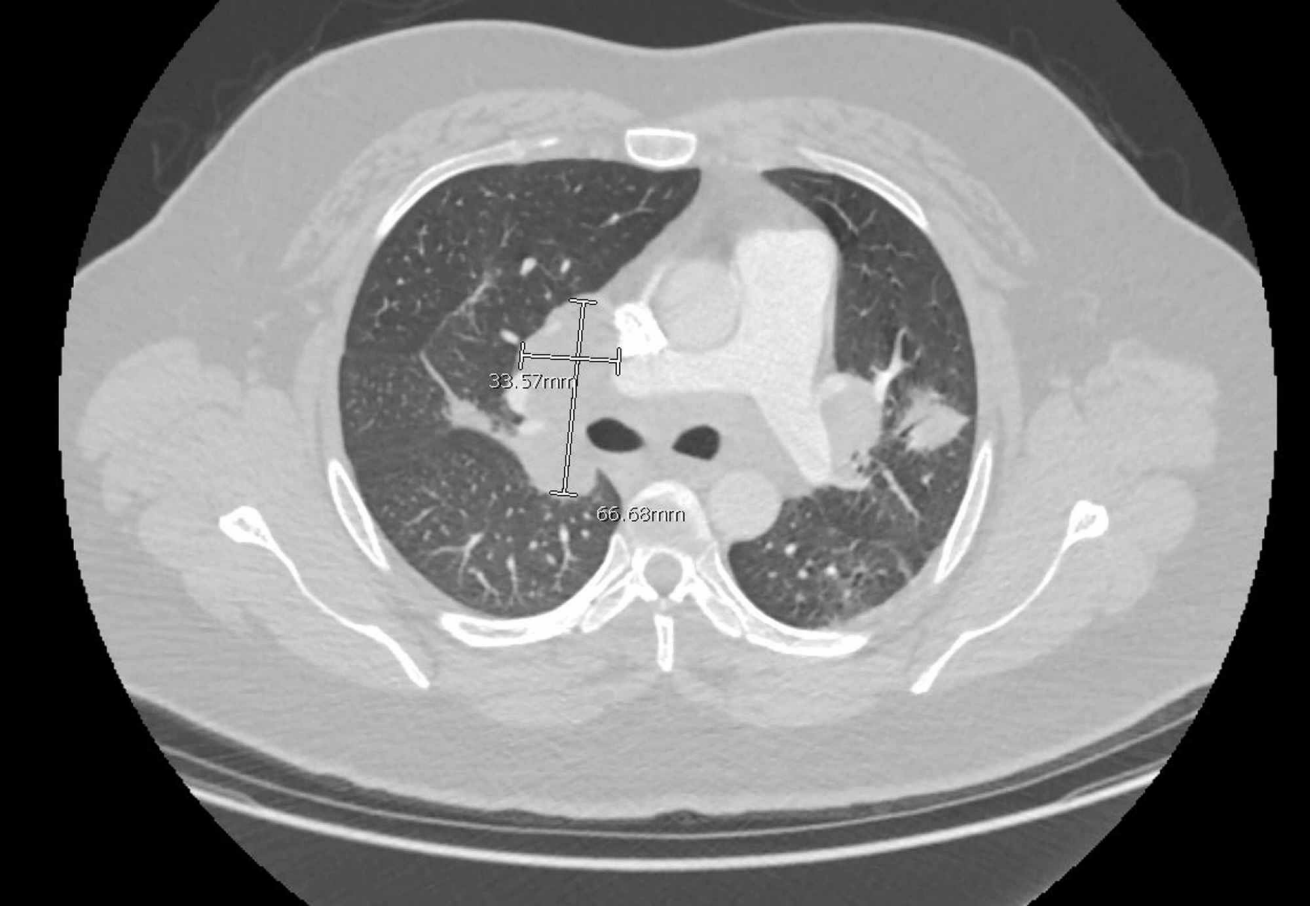
CT angiography chest transverse view showing extensively enlarged pathological hilar lymph nodes measuring 3.4 cm × 6.7 cm (dimensions marked in white).

**Figure 6:**
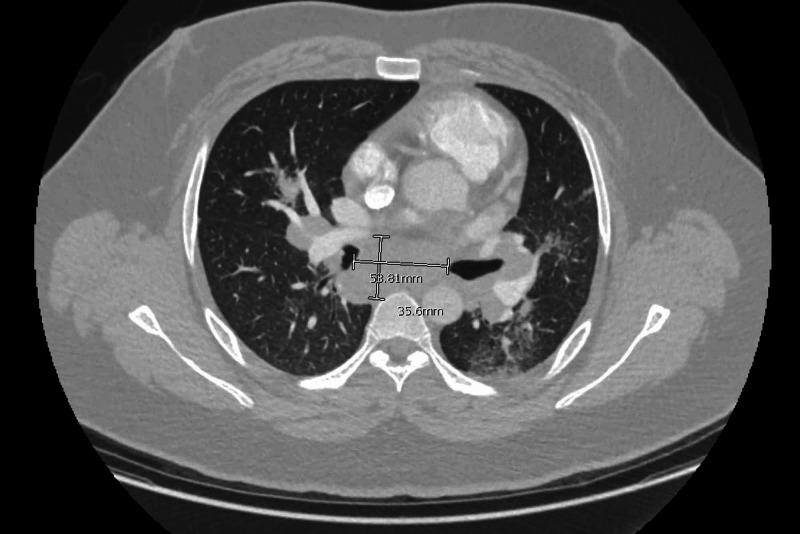
CT angiography chest transverse view showing enlarged subcarinal lymph node measuring 3.7 cm × 5.4 cm (dimensions marked in white).

This was followed up by a CT chest, which revealed bilateral infiltrates and enlarged hilar, anteroposterior, and subcarinal lymph nodes (Figures 3-6).

**Figure 7:**
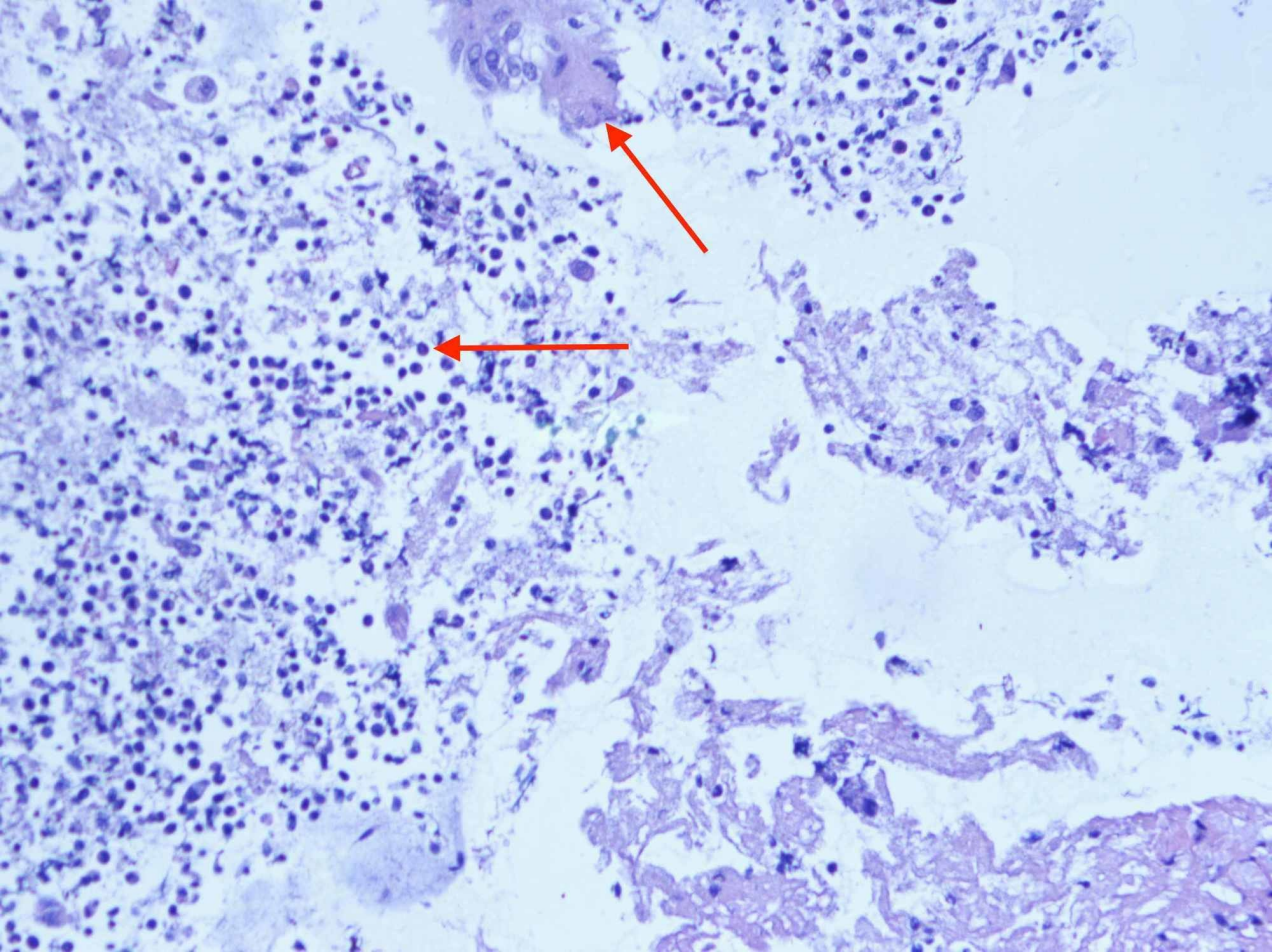
Fine needle aspiration cytology of subcarinal lymph node showing necrotic debris and lymphocytes (marked in red).

**Figure 8:**
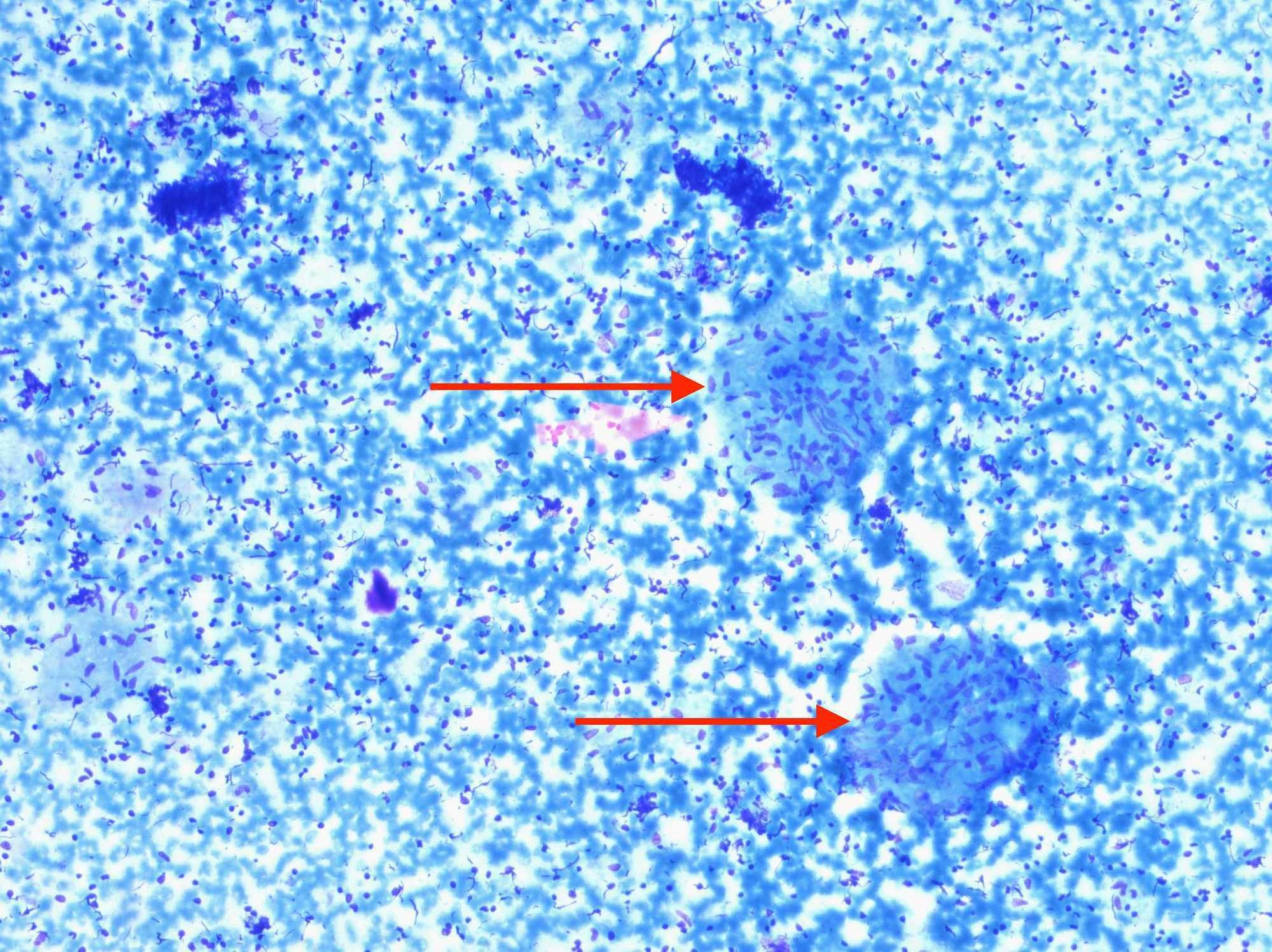
Fine needle aspiration cytology of subcarinal lymph node showing epithelioid histiocytes (marked in red).

The patient was discharged home and advised to follow up as an outpatient (OP) to further evaluate the stated imaging findings. On follow-up, the patient remained asymptomatic. On further history taking during the OP visit, he denied any history of recent travel, exposure to occupational hazards, asbestos, silica or beryllium, cow/dog/cat hay or fur, radiations, and chemotherapy. Family history was significant for sarcoidosis in paternal aunt. Review of systems was negative for cough, hemoptysis, night sweats, fever, chills, unintentional weight loss, or any rashes. On physical examination, the patient had normal vitals; the lungs were clear to auscultation with no palpable lymph nodes. The patient’s laboratory workup was significant for mild thrombocytopenia (132 k/cumm) and low 25-hydroxy vitamin D levels (7 ng/mL), mildly elevated lactate dehydrogenase (294 units/L), and angiotensin-converting enzyme (ACE, 126 units/L). Complete blood count, comprehensive metabolic panel, anti-neutrophil cytoplasmic antibody panel, interferon-gamma, Histoplasma, Blastomyces antigen, HIV 1/2 antibody, and hepatitis C were negative. Pulmonology was consulted. The patient mentioned having occasional chest pain, non-pleuritic, and unrelated to exertion on a follow-up visit. Further workup for infectious and autoimmune diseases, including anti-cyclic citrullinated peptide, rheumatoid factor, and Aspergillus antigen/antibody, was negative. However, IgE (126 kU/L) was mildly elevated. Pulmonary function tests (PFTs) showed a mixed obstructive and restrictive pattern. On repeat imaging three months later, no change in lymph node and spleen size was noticed. This workup was followed by an endobronchial ultrasound with endobronchial needle aspiration. Bronchoalveolar lavage (BAL) fluid revealed high neutrophil count (80%), normal lymphocytes (10%), low monocytes/macrophages (9%), and high eosinophil counts (1%). Fluid cultures were negative for fungal, acid-fast bacilli (AFB), and other respiratory pathogens. Cytology report showed benign respiratory epithelial cells, macrophages, and mixed inflammatory cells, with no atypical cells. Fine needle aspiration of subcarinal lymph nodes revealed necrotizing granulomatous inflammatory appearance, which in correlation with the absence of atypical cells and negative infectious and autoimmune workup, is consistent with a diagnosis of necrotizing sarcoidosis (Figures 7, 8).

The patient was started on prednisone with the plan to taper it subsequently and monitor for radiological improvement on repeat CT scans.

## Discussion

Research studies predict that the number of hospital admissions in patients with sarcoidosis is higher than those without it [7]. Therefore, the disease poses a significant financial burden on the United States healthcare industry. Approximately 20% of these patients eventually end up having lung-related complications [2]. Based on our literature review, it remains unclear if NSG is a separate entity or a variant of classic nodular sarcoidosis [8]. Studies predict that it is a later presentation or necrotic variant of classic sarcoidosis. Hence, it can be named as sarcoidosis with an NSG pattern [9]. The clinical and radiographic presentation of the two entities is very similar, as evidenced by a female predominance and a lack of difference in the incidence of pulmonary and extrapulmonary symptoms. NSG is more common in Caucasians than African Americans; the median age is 42 years compared with 20-39 years in the case of classic sarcoidosis [10], with a higher frequency of intrathoracic lymphadenopathy in the classic variety. The patient described in our case study had features suggestive of both classic and necrotizing sarcoidosis: an elevated ACE level, intrathoracic lymphadenopathy, and necrosis within granulomas on histology. Sarcoidosis has traditionally been associated with a non-necrotizing histological pattern; our case study shows that necrosis on histology might not necessarily rule out the diagnosis. NSG should be kept in the differential of necrotizing granulomatous process. Lymphoma might commonly be confused with NSG due to an extensive lymph node involvement. Hence, NSG’s definite diagnosis requires excluding a malignant, infectious, or autoimmune process that might present with granulomas. NSG holds a favorable prognosis with early initiation of steroid therapy leading to positive outcomes [11].

Further studies are needed to see whether there is a difference in mortality and/or morbidity with early versus a delayed treatment and the effectiveness of various treatment regimens in preventing relapse. Hence, this study focuses on a disease that needs extensive research in different population groups worldwide to establish definite diagnostic criteria and etiology, possible genetic and environmental association, and brings up rare presentations. This will possibly pave the way for early diagnosis of the disease, and hence, efficient use of health-related resources.

## Conclusions

NSG is a rare entity that should remain in the differential diagnoses of necrotizing granulomatous processes of the lung. Due to its indolent course, it might remain elusive to medical professionals. A high degree of suspicion is implied. Knowing the varied presentations of this multifaceted disease might lead to the delivery of timely and cost-effective care to the patients, thus providing high-value care. Early initiation of steroids, ruling out lymphoma in the workup, might be helpful. Further studies are needed to explore its disease diversity, and genetic and epigenetic predispositions. 
